# In-Vivo Microsystems: A Review

**DOI:** 10.3390/s20174953

**Published:** 2020-09-01

**Authors:** Paddy French

**Affiliations:** Laboratory for Bioelectronics, Faculty of Electrical Engineering, Mathematics and Computer Science, TU Delft, Mekelweg 4, 2628CD Delft, The Netherlands; p.j.french@tudelft.nl; Tel.: +31-15-2784729

**Keywords:** medical sensors, implants, in-vivo devices, microsystems

## Abstract

*In-vivo* sensors yield valuable medical information by measuring directly on the living tissue of a patient. These devices can be surface or implant devices. Electrical activity in the body, from organs or muscles can be measured using surface electrodes. For short term internal devices, catheters are used. These include cardiac catheter (in blood vessels) and bladder catheters. Due to the size and shape of the catheters, silicon devices provided an excellent solution for sensors. Since many cardiac catheters are disposable, the high volume has led to lower prices of the silicon sensors. Many catheters use a single sensor, but silicon offers the opportunity to have multi sensors in a single catheter, while maintaining small size. The cardiac catheter is usually inserted for a maximum of 72 h. Some devices may be used for a short-to-medium period to monitor parameters after an operation or injury (1–4 weeks). Increasingly, sensing, and actuating, devices are being applied to longer term implants for monitoring a range of parameters for chronic conditions. Devices for longer term implantation presented additional challenges due to the harshness of the environment and the stricter regulations for biocompatibility and safety. This paper will examine the three main areas of application for in-vivo devices: surface devices and short/medium-term and long-term implants. The issues of biocompatibility and safety will be discussed.

## 1. Introduction

The importance of in-vivo measurements is that these are performed directly on the living tissue, either as surface or internal devices. The miniaturization of the devices, and the system (sensor, electronics, power and communication) has led to many more applications, making them practical to be used as internal devices.

The term in-vivo comes from the Latin “within the living”, although surface devices are usually included, when we discuss in-vivo. Early examples of in-vivo devices include false teeth made from shells by the Mayan people around 600 AD. These devices along with bone implants were not intended to measure, but to restore function and/or support the hard tissue during recovery. Today there are many in-vivo devices used for measuring and stimulation.

During the 20th century the development of sensors led to new in-vivo applications. In 1938, Nims and Marshall studied the effects of salts acids, adrenaline and dextrose on the blood pH in anesthetized dogs and cats using in-vivo devices [1,2]. This was an early example of in-vivo sensors. Since then the in-vivo possibilities have expanded greatly and a wide range of devices are used on human patients.

Electrical signals measured on the skin can give valuable information about the condition of the patient. Early examples, still widely used today, are electrodes for electrical measurements of the heart electrocardiogram (ECG) [3,4]. These early devices were simple electrodes, but many developments have been made by integrating electronics into the electrodes to improve the signal-to-noise ratio (SNR) [5,6,7]. Today there are many examples of skin devices with sensors for measuring a range of parameters [8,9]. Simple patches can be used to measure parameters such as temperature, heart rate, glucose, blood pressure, etc. Some devices have also been developed to measure glucose in the tissue, just under the epidermis. These can be considered as surface devices, even though some are designed to pierce the epidermis.

Entering into the body for the short term we have the catheters, which are generally used for up to a maximum of 72 h. Bladder catheters date back to 3000 BC in Egypt, and are widely used today mainly to drain or inject fluid [10]. Cardiac catheters are used in the blood vessels where they can be used for measurement or treatment. Early examples here were pressure sensors and balloon catheters for the treatment of stenosis or aneurysm. These techniques are still widely used today [11,12]. Using silicon there is also the option to build in more sensors and more functionality [13].

Medium term devices can be used for monitoring after an operation. These could be used for a few days, up to a month. It is important that the device can be removed within a month to avoid the increased regulations for biocompatibility (which will be discussed below). Longer term implants have to comply to many more safety and biocompatibility tests. In general, the regulations for biocompatibility and safety depend on the use and the length of time inside the body and where in the body the device is to be used. Internal devices implanted for more than 30 days have to pass the greatest number of tests.

Probably the best known of implanted devices is the pacemaker, mentioned above. In the 19th century scientist began to understand the electrical operation of the heart [14] and this understanding eventually led to the pacemaker. The first pacemaker to be implanted was in 1958 at the Karolinska Institute in Solna, Sweden [15]. Although early device had some complications, they were able to extend the life of the patients. Significant improvements have since been made and pacemakers are now widely used and save many lives around the world. Tests of implanted artificial hearts were performed in 1969. The hearts performed well mechanically, but all devices resulted in adverse body reactions.

The main operation of the pacemaker is to stimulate and regulate the heart [15,16,17]. Increasingly, sensors and improved electronics are being used to optimize the efficiency of the devices [18,19]. These improvements allow the pacemaker to optimize the actions to fit the needs of the user. Furthermore, the lifetime of the devices has been significantly extended through improved battery technology and reduction in energy requirement. Although the pacemaker may not be considered a microsystem, it contains a number of microsystems to ensure correct and optimal functioning.

Other long-term implants include cochlear and retinal implants [20] and deep brain stimulators. These different approaches, applications and devices will be discussed in the sections below.

## 2. Biocompatibility and Safety

Safety and biocompatibility are essential considerations for in-vivo devices and in particular implants. Biocompatibility is defined as: the ability of a medical device to function and exist in an in-vivo environment for a given period of time, with NO detrimental effect on the host. The safety of a device is defined as: “the state of being certain that adverse effects will not be caused by some agent under defined conditions”. These are essential starting points for the development of any in-vivo device.

The first mechanical hearts implanted into patients all failed due to the body’s reaction and not from mechanical failure. Table 1 [21], shows the tests required on in-vivo devices to obtain approval. The FDA (Food and Drug Administration, Silver Spring, MA, USA) only gives approval for complete devices for specific applications. Using materials already used for similar applications will simplify the process, but is still no guarantee of approval. The devices need to survive the sterilization process. For disposables, this only needs to be once, but reusable will have to survive this process many times. The main sterilization techniques are moist heat sterilization, ethylene oxide gas sterilization and radiation sterilization.

The main effects of the materials on the host are:

- Toxicity;
- Injury;
- Inflammation;
- Infection;
- Tumorigenesis.

Furthermore, the device has to survive the harsh biological environment. An overview of biomaterials and the issues of biocompatibility can be found in [22].

**Figure 1:**
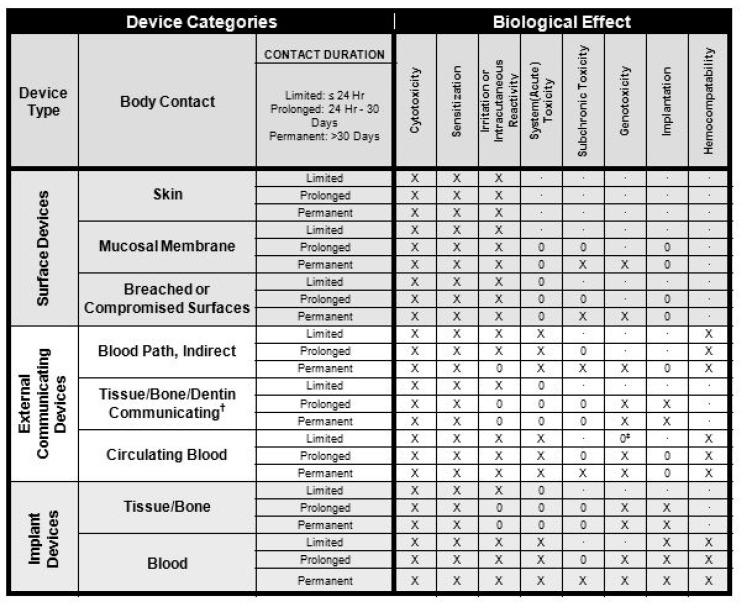
Overview of biocompatibility tests required for Food and Drug Administration (FDA) approval for use on patients. X: ISO 10993-1 test; O: additional test that may be applicable for some applications; †: tissue includes tissue fluids and subcutaneous spaces; ‡: for all devices used in extra-corporeal circuits.

In Figure 1 cytotoxicity is defined as being toxic to cells, such as a venom. Further tests include sensitization and irritation. These are required for all devices in contact with living cells. As the length of time of contact increases, the test requirements for toxicity are increased. Genotoxicity means that the devices implanted cause a change in the genetic information in cells causing mutations. The hemocompatibility is to ensure that the body does not form visual thrombus material on the surface. This can cause problems to the host and may also lead to malfunction of the device.

In many cases the solution is to coat the sensor system with a suitable material. A range of polymers (both natural and synthetic) has been used as coating/encapsulation. A number of metals are also used, such as stainless steel, cobalt and titanium alloys.

In addition to protecting the host from unwanted effects, it is important to ensure the correct operation of the device for the required period. For example, cochlear implants contain active electrodes operating in a salt solution. This creates an electrochemical cell that, over time, results in the degradation of the electrodes. For long term implants, and in particular optical devices, the signal can be greatly diminished by deposition of cells on the surface. Furthermore, the device should not have any sharp corners or cavities in which bacteria can settle and also no extruding parts, which can break off and become loose in the body.

In terms of approval, there is no universal biocompatible material, it is dependent upon where in/or on the body and how long the contact is maintained.

## 3. Examples of Important Parameters

There is a wide range of parameters where in-vivo devices can give valuable information. Where possible these are measured using surface devices, but in some cases a reliable measurement can only be achieved by measuring inside the body. From the surface we can perform electrical, temperature, heart rate, blood pressure, oxygen saturation, etc., measurements. There are also some examples of glucose and lactate measurements with surface devices. For the surface devices the regulations are less strict and the procedure simpler. Implants can measure directly in the tissue or in the blood, which in some cases yields faster and more reliable measurements. Glucose is an example where both surface and implanted devices have been developed. With 422 million diabetics worldwide in 2014 and rising numbers, glucose measurement has become a major issue. Traditionally, a small blood sample was taken and analyzed. It is preferable if no sample is needed and no active participation from the patient is required. More recent developments include surface devices, either with or without microneedles [23]. These surface devices can connect to a display to keep the user informed on blood sugar levels and the need for additional insulin. Complete implanted devices are preferable for the user, but extra care needs to be taken to ensure stability and safety [24,25]. Although blood and tissue render the best and fastest measurement, examples can be found for measurement in the eye, saliva and bladder [26,27,28]. In addition, there are many other parameters that can be measured in different ways. In addition, parameters such as lactate, and pH can give valuable information about the patient. These will also be dealt with below.

## 4. Surface Devices

**Figure 2:**
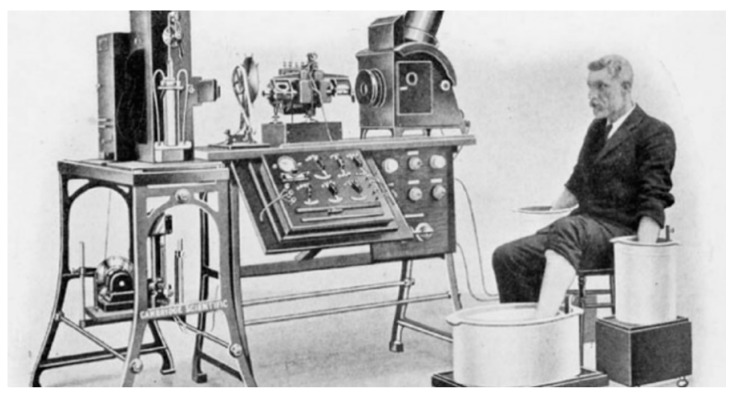
Image of the electrocardiogram (ECG) demonstration of Einthoven, around 1911 [29].

One of the well-used group of devices is the simple electrode. These can be used for systems such as electroencephalogram (EEG), electrocardiogram (ECG), electromyogram (EMG), mechanomyogram (MMG), electrooculography (EOG) and galvanic skin response (GSR). Since the 17th century there has been interest in the link between living beings and electricity. Galvani showed the link between muscle contraction and electrical impulses and in 1887 British physiologist Augustus D. Waller of St Mary’s Medical School, London published the first human electrocardiogram [4]. This was recorded with a capillary electrometer from Thomas Goswell (a technician in the laboratory). In 1889 the Dutch physiologist William Einthoven demonstrated ECG measurements [4,29], as shown in Figure 2.

Today ECG electrodes connected with the use of a gel to improve the electric connection and in some cases local electronics can improve performance [5].

**Figure 3:**
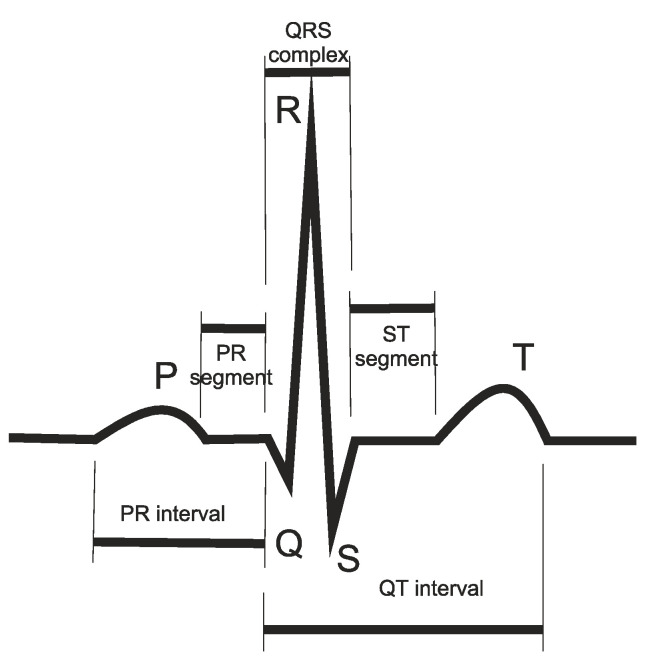
Typical ECG measurement.

Taking the example of an ECG measurement, shown in Figure 3, the shape and regularity of this signal, from beat-to-beat gives valuable information to the doctors about the health and functioning of the heart.

Other devices include an electrode for prostheses. The use of prostheses goes back 3000 years. Hand prostheses were originally simple hocks or wooden appendages. Later, prostheses were developed where the user could perform some movement using simple surface electrodes. Using more electrodes, greater dexterity can be achieved [5]. There is also increasing interest in implanting the electrodes and interacting directly with the nerves [6]. A more complex interaction with the patient’s nervous system may eventually lead to prostheses with similar operation and dexterity as the original.

**Figure 4:**
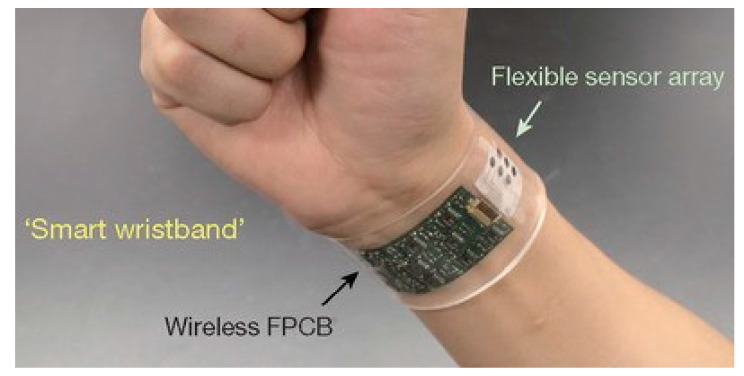
Example of a skin device with sensors [30].

With the development of flexible microsystems there has been a great increase in wearable devices [30,31]. Wearable devices can be in the form of a plaster or arm band, or be built into clothing. These devices can monitor a range of parameters such as heart rate, temperature, blood pressure, etc. In addition, surface devices can be used to measure sweat. The chemical constitution of sweat can reveal much about the health and physical condition of the user, such as the onset of muscle fatigue. Most of these devices need to be flexible, waterproof and in many cases stretchable. Such an example is given in Figure 4.

**Figure 5:**
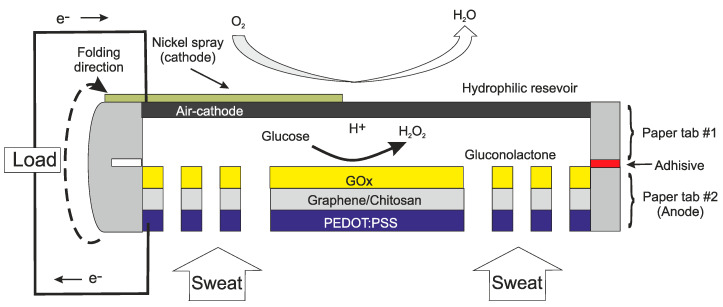
Cross section of the self-powered sensor for measuring glucose in sweat [36].

Another important example of a surface device is to measure glucose for diabetics. Although glucose in blood reacts more quickly to changes in the body, skin electrodes can yield a good tacking of blood sugar levels. Implantable devices for glucose will be discussed later. Sweat and surface electrode measurements have also been made for parameters such as lactate [32,33], ammonia [34] and glucose [35,36]. The device in [36] has the additional feature of being self-powered. The device is not only measuring the glucose, it is using the glucose to power a fuel cell. The structure of this device is given in Figure 5.

With skin electrodes, the measurement volume can be just under the epidermis [37]. Another type of device is to use electromagnetic patches to measure the intracranial fluid-volume shift [38].

Devices that pierce the epidermis but remain in the dermis can also be considered as surface devices. Many of these are used for inserting or extracting fluid through microneedles [39,40]. However, there are examples of using microneedles for sensing [41]. The microneedles can be fabricated on a range of substrates by a number of techniques. These include, etching, molding and more recently 3-D printing [42]. There is a range of materials that can be used, depending on the application, while ensuring that the material does not induce any irritation. The basic structural materials used include metals, silicon and polymers (such as SU8).

## 5. Short-Medium Term Internal Devices

The most prominent short-term device is the catheter. There are many types of catheters, some of which include microsensors. The cardiac catheter is brought into the body through blood vessels and the tip is brought to the place of interest for measurement or treatment. Pressure sensors for catheters were one of the first mass markets for silicon sensors. These devices were disposables, which led to a large volume and thus lower prices. Other devices are short term implants for monitoring after an operation. These devices are usually placed where they can be easily removed after the required period. One example, described below, is to monitor tissue vitality around a wound. This gives important information about the healing process.

### 5.1. Catheter Devices

**Figure 6:**
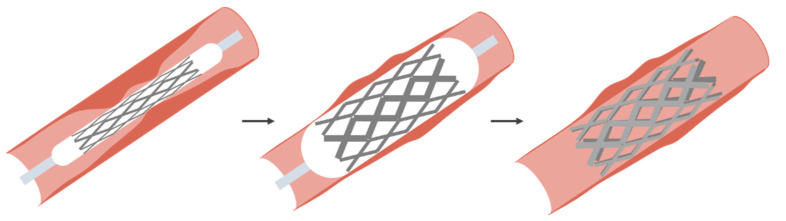
Basic process for treating a stenosis using a balloon catheter [45].

The catheter dates back to ancient China, Rome and Egypt. These were simple bladder catheters. Cardiac catheters date back to Stephen Hales (1677–1761) and Claude Bernard (1813–1878), both using them in animals. The first example in humans was by Werner Forssmann in 1929 on himself [43,44]. Cournand and Richards continued the work and the three eventually received the Nobel prize for medicine in 1959. The expansion of measurement and treatment using catheters came with the work of Dotter [12]. Dotter developed the method for treating stenosis (narrowing of a blood vessel) and later aneurism (expansion of a blood vessel). The basic technique is to use a wire mesh on a catheter. The catheter is equipped with a balloon, which can be activated by the surgeon when in the location of the stenosis. This basic process is shown in Figure 6. Here the balloon is expanded to push the mesh against the sidewall and then deflated and removed. In the case of an aneurism, the wire mesh is embedded in a Teflon layer, which will seal off the aneurism. Once the stent is in place, the pressure on the weakened blood vessel wall is removed and the stent forms a new blood vessel.

**Figure 7:**
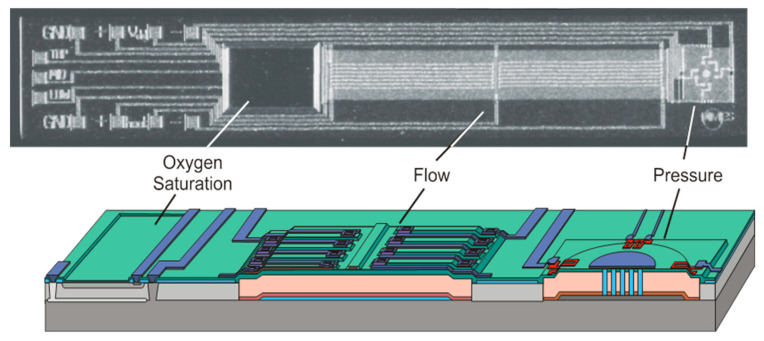
Example of a multisensor designed for cardiac catheters [15].

For silicon devices, and early application in catheters was the pressure sensor, which is still widely used today. These devices could be made small enough to fit a wide range of catheters, but these were all a single sensor. In general, these were simple membranes with piezoresistive read-out and wires leading through the catheter to the instrumentation outside. One advantage of using silicon is not only the ability to make small devices, but also more devices can be included on a single chip. One such example is to include pressure, flow, oxygen saturation and temperature on a single chip. This is shown in Figure 7 [13].

Here the pressure sensor is a simple membrane, the flow sensor uses thermal techniques and the oxygen saturation device measures color of the blood by probing its absorbance, commonly defined as the spectral ratio between oxy- and deoxy-hemoglobin, using red and near infrared. The length of the chip was 7 mm, which is suitable for larger blood vessels. For each sensor it is necessary to match the length to the required flexibility (or make the sensor flexible) and a width to fit the catheter. The pressure sensor remains an important device for catheters. For some applications, these have been further miniaturized for smaller catheters. Additional features have also been considered, such as automatic compensation for respiration during catheterization [46].

Another example is using electrodes to measure blood impedance. From this, blood viscosity can be estimated, by measuring impedance, using four rings on the catheter, at two frequencies [47]. Silicon technology allows a wide range of possibilities for sensing, although, as discussed above, appropriate coating layers and packaging are required.

**Figure 8:**
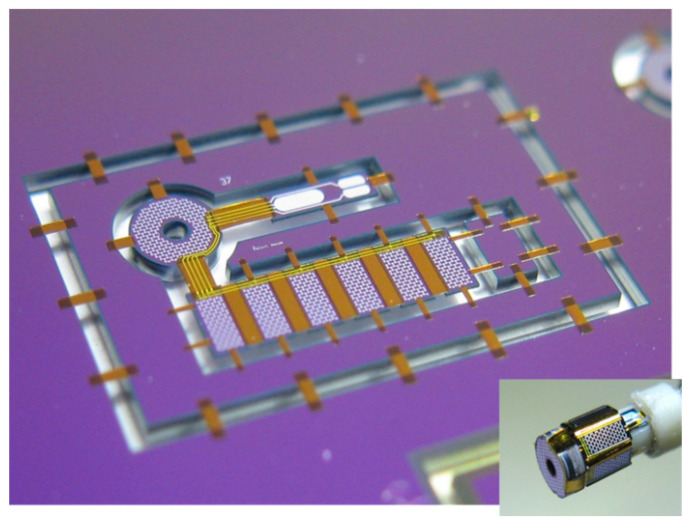
Complete ultrasound device, before release from the substrate and (insert) the final device. Reproduced with the kind permission of Prof. dr. Ronald Dekker, TU Delft, The Netherlands (private communication).

Ultrasound is a valuable tool in medicine. A device for monitoring that can now be fitted to a catheter, or even a guidewire, is a miniaturized ultrasound device. This allows the cross section of the blood vessel to be seen and also can give information about the material of any build-up on the sidewall. These devices use micro-ultrasound devices, usually using CMUTs (capacitive micromachined ultrasonic transducers) or piezoelectric elements for both transmitting and receiving. This is of particular interest with the build-up of a stenosis. The use of flexible materials deposited on a silicon wafer followed by micromachining techniques to release the devices has led to many new devices for implants and catheters [48,49,50]. An example of such a device, before removal from the silicon substrate, is shown in Figure 8. The device is held onto the chip using thin polymer bridges. These are later removed using laser cutting. This same thin polymer layers serve as the hinges to wrap the devices around the guidewire. The thicker areas, which are rigid, contain the ultrasound devices. The finished device, on the guide wire is shown in the insert. The thin flexible parts act as the hinges and the rigid parts hold the ultrasound devices.

**Figure 9:**
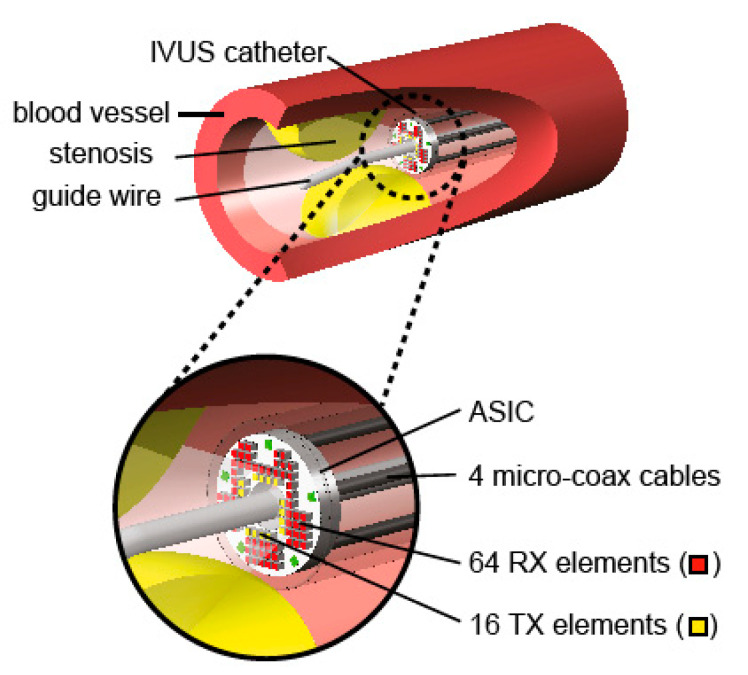
Example of an ultrasound probe in a blood vessel [51]. Reproduced with the kind permission of Dr. Michiel Pertijs, TU Delft, The Netherlands.

These types of devices allow measurements to be performed directly in the blood vessels as shown in Figure 9. This device is designed to look forward in the blood vessel to measure a stenosis. This will give information on the extent of the stenosis and also information about the material of the stenosis.

Similar devices, developed by the same group, have been used for studies in the heart via the esophagus [52].

### 5.2. Minimally Invasive/Laparoscopic Surgery

Laparoscopic surgery enables the operation to be performed with minimal damage to healthy tissue. Here, as with catheterization, the surgeons direct view and feedback is lost. Probes, with cameras can give visual feedback, in many cases, but a range of sensors can be used to give improved feedback. Although only widely used in more recently the first know use of laparoscopic techniques dates back to 1901 when Georg Kellin, in Germany performed this approach on a dog and later in 1910, Hans Chritian Jacobaeus, Sweden, performed this operation on a human [53]. Important sensors include tactile and slip sensors [54,55,56]. These are important to ensure the tissue is firmly held, without causing any damage. Systems have also been developed with haptic feedback systems, with a range of sensors, for training [57].

### 5.3. Medium Term Implants for Monitoring

There can be great benefit from monitoring in the days after an operation. However, the device needs to be either removed, or ejected by the body within 30 days to avoid regulations for permanent implants. One such example is a device for measuring tissue vitality of the colon after a colon operation. Tissue vitality is the health of the tissue, which is an indication of how well the tissue around anastomosis is healing. During the operation the device can be fixed to the tissue on the inside of the colon. Using dissolvable sutures, the device will be ejected by the body after about 10 days, which covers the critical period. If the anastomosis is not successful, leakage may occur allowing *Escherichia. coli* (bacteria present in the colon) to leak into the body resulting, potentially, in total organ failure. To assess tissue vitality, we can measure oxygen and carbon dioxide dissolved in the tissue and temperature. Temperature measurement gives an indication of local infection, oxygen measurement shows that the cells are being fed with oxygen through the blood system and the carbon dioxide, as a waste product of the cells, shows that the cells are functioning correctly. The dissolved oxygen can be measured using fluorescence quenching in a polymer on the chip. The polymer is impregnated with ruthenium, which when illuminated with blue light will fluoresce with red light. If oxygen is present, this kills (quenches) the fluorescence. The higher the oxygen concentration, the faster the fluorescence is quenched. Therefore, by measuring the fluorescence decay, the oxygen partial pressure can be calculated. Carbon dioxide can be detected using fluorescence intensity using a different impregnated particle. Temperature can be measured using a number of devices available in silicon technology, such as a p–n junction.

**Figure 10:**
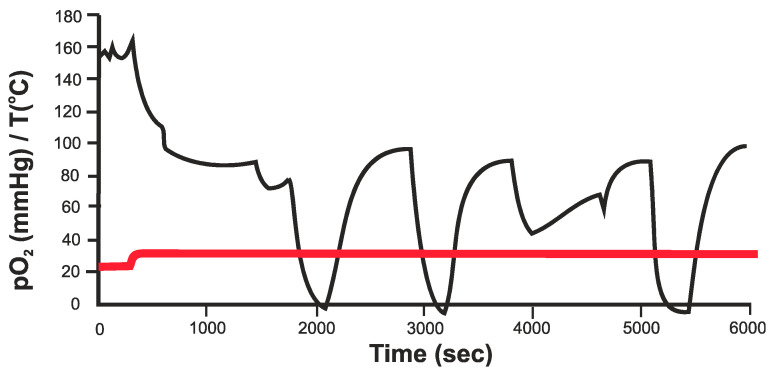
pO_2_ measurements in the kidney, where the arterial blood supply is briefly interrupted [58].

An example of measurements of O_2_ in the kidney is given in Figure 10. Although not completely calibrated, this shows a direct response to a reduction in oxygen supply [58]. The initial measurement is in air. Once in contact with the tissue the pO_2_ reaches a stable level. During these measurements the main artery to the kidney was briefly interrupted and the oxygen in the main tissue measured. Measurements were also performed on the colon and showed the stability of the device during the required period.

**Figure 11:**
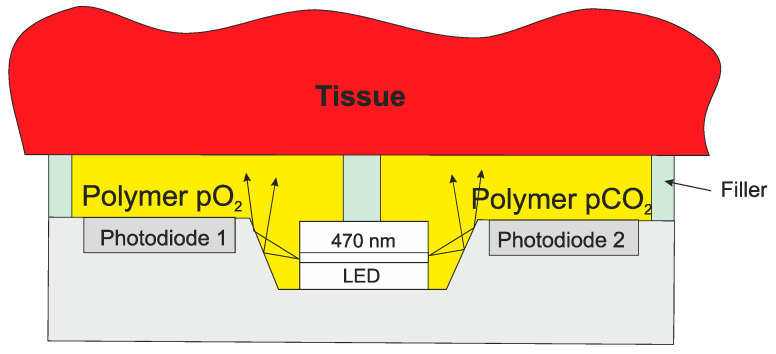
Integrated O_2_/CO_2_ sensor for tissue measurements.

The whole system can be incorporated into a single chip with a LED fixed into the chip as shown in Figure 11. Both O_2_ and CO_2_ can be measured using the same excitation wavelength, although the additives to the polymer are different. A similar technique can also be used for a wide range on measurements of wounds, including burns.

As mentioned above, the surgeons want to avoid a second operation to remove the implant. In the case of the colon device (fixed to the inside of the colon with dissolvable sutures), the device will be ejected out of the body after about 10–15 days. Small devices under the skin could be removed with a minor incision, but recent materials advances allow devices to be made that will be absorbed by the body. The development of organic semiconductors/conductors and porous materials means that fully operational devices can be implanted that will be absorbed into the body [59,60,61]. Although the performance will not be as high as conventional semiconductor devices, they may be sufficient for the application. As long as this time is longer than the required monitoring period, and shorter than the 30 days where the stricter regulations come into force, a post-operative monitor can be made.

## 6. Long Term Implants

The Mayan people developed the idea of using shells as teeth replacements. Later, implants were used to support bones, where the body could not repair the damage and the implant would support and stimulate healing. These early examples were mainly for mechanical purposes. An early example of electrically active implants in the body is the pacemaker. Others include cochlear and retinal implants, and deep brain stimulators.

### 6.1. Pacemaker

**Figure 12:**
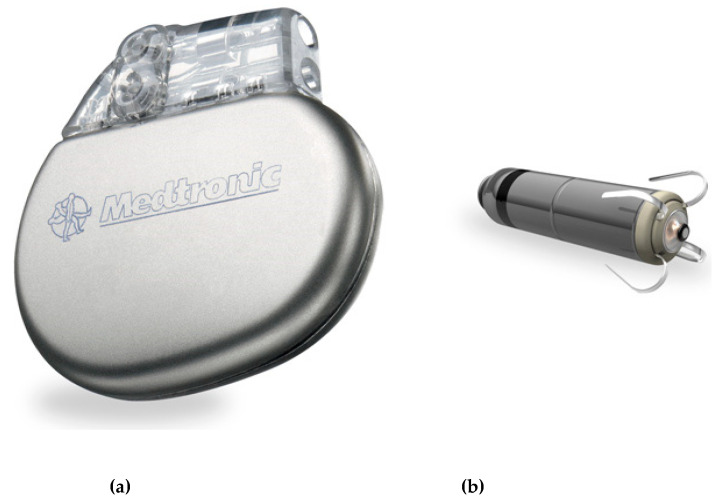
Modern pacemaker implanted in the chest (**a**) and using cables to the heart and new generation of pacemakers for complete insertion in the heart (**b**). © Medtronic (not the same scale).

The name pacemaker is a general term, but there are many different types depending on the need of the patient. They are used regulating the heart such as pacing, defibrillation and synchronization. The first real understanding of the electrical significance came in the 19th century, and through the first half of the 20th century, there were many developments that eventually led to the pacemakers we have today [62,63,64,65]. Early devices were external and although inconvenient to wear, it was easy and straightforward to replace them. With fully implanted devices the battery and efficiency of the device was crucial. Developments in battery technology and improvements in the efficiency of the devices has led to great reduction in size and increased lifetimes. Modern pacemakers are implanted in the tissue of the chest. A cable is led from there into a blood vessel and then into the heart. The tip is anchored into the tissue of the heart. Future pacemakers will be much smaller and potentially the whole device, including battery, can be inserted into the heart using minimally invasive surgery. A modern pacemaker and the future miniaturized pacemaker are shown in Figure 12. The electronics in the modern pacemaker is designed to optimize the signals produced and minimize power consumption [19].

The pacemaker is much more than a simple stimulating device. Increasing numbers of sensors are used to enable the device to work optimally and best serve the user. A number of measurements can be fed back to the pacemaker to enable to best choice of action to be taken. Furthermore, it is possible to communicate with the pacemaker during a check-up and adjust the settings if necessary. This approach has great advantages for the patient. However, this also requires additional security to protect the system from unwanted intrusion [66]. Sensors such as pressure, oxygen saturation in addition to ECG can be used to optimize the operation of the system [67,68].

### 6.2. Cochlear Implant

The cochlear implant is a device that stimulates the nerves in the cochlea to restore hearing. The basic cochlear implant was invented in 1957 by André Djourno and Charles Eyriès [69]. These were further developed in the 1960s and have restored hearing to many deaf people around the world.

**Figure 13:**
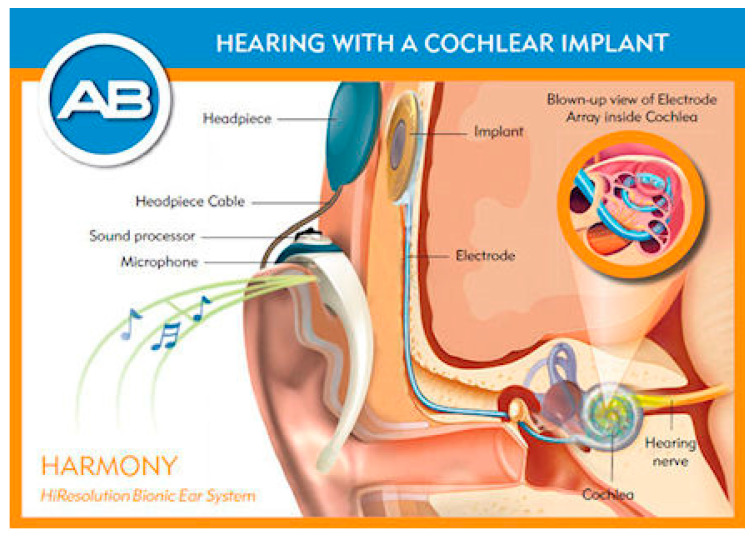
Modern cochlear implant for restoring hearing to the deaf [48]. © Advanced Bionics.

**Figure 14:**
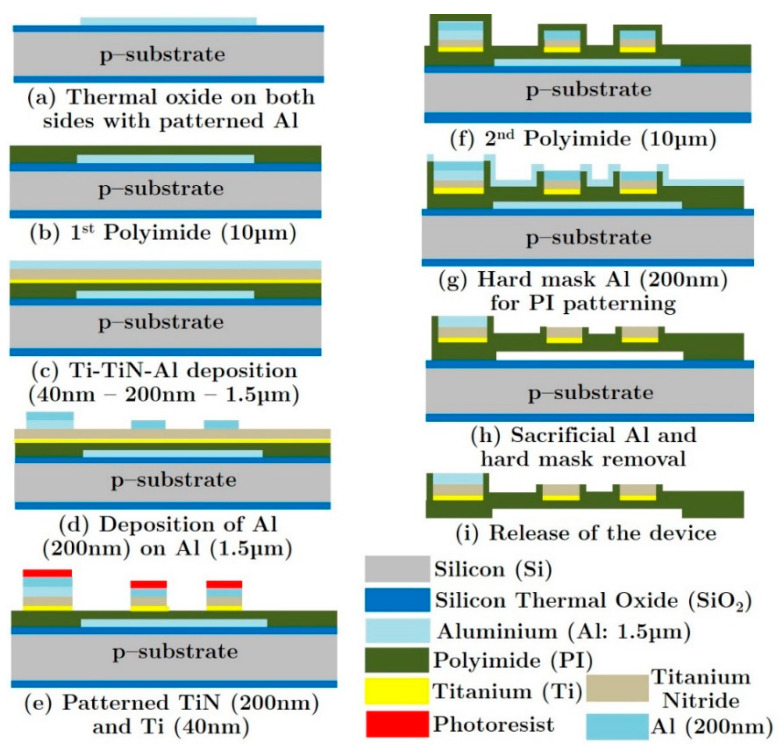
Silicon based process for a new cochlear implant using silicon processing and polyimide [70].

The basic set-up for the cochlear implant is given in Figure 13 [70]. The sound is received by a microphone attached behind the ear. The signal is transmitted to a unit inside the body where it is processed into the desired format and sent to the implant in the cochlea. The electrodes then stimulate the nerve electrically and the signal is then further processed by the body. The limitations of all cochlear implants at the moment are the number of electrodes (max 22) and the depth of penetration into the cochlea (about 1 ½ turns) resulting in the non-detection of the lower frequencies. There has been some research using mechanical or optical stimulation or connecting directly to the auditory nerve, but this has not yet led to commercial devices [71,72]. The quality of the sound detected and the frequency range can be improved using IC technology. This will allow more electrodes to be used and also reduce the size of the tip to increase the depth of penetration. One proposed approach involves using polymers on top of silicon and releasing the polymer structure from the silicon to generate a flexible polymer-based structure. This basic process is given in Figure 14 [70]. Polymers are deposited onto a silicon substrate along with the metallization. At the end of the process the polymer (with electrodes) is removed from the silicon to yield a flexible probe. In this case TiN was used as an electrode, in place of platinum, since evidence has shown that in the thin film form, TiN is more stable in this harsh environment. An extension of this idea could be to leave a thin layer of silicon and include some electronics in the probe. This would allow some local multiplexing to reduce the number of metal tracks required and, therefore, enable the inclusion of more electrodes.

In addition to the development of the probe itself, it is important to optimize the signal. The implanted low-power electronics converts the incoming signal to be transmitted to the electrodes in the probe [73].

### 6.3. Retinal Implant

**Figure 15:**
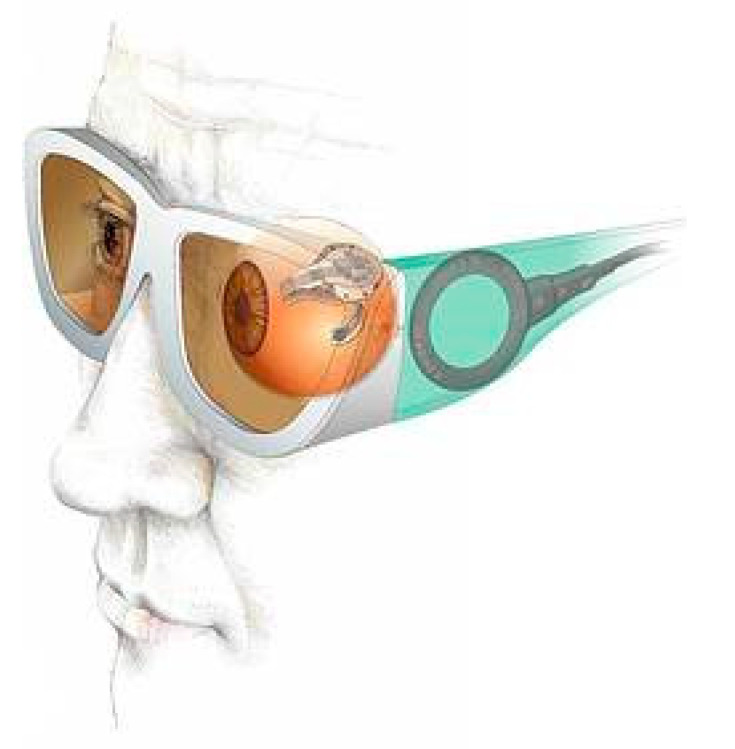
General layout of the retinal implant [74].

The retinal implant is not as far as the cochlear implant in terms of commercialization, but shows great potential in restoring sight to the blind. In a similar fashion to the cochlear implant, electrodes are used to stimulate the nerves. The basic structure of the system is given in Figure 15. This device was developed by Intelligent Medical Implants AG (IMI) in Switzerland [74].

**Figure 16:**
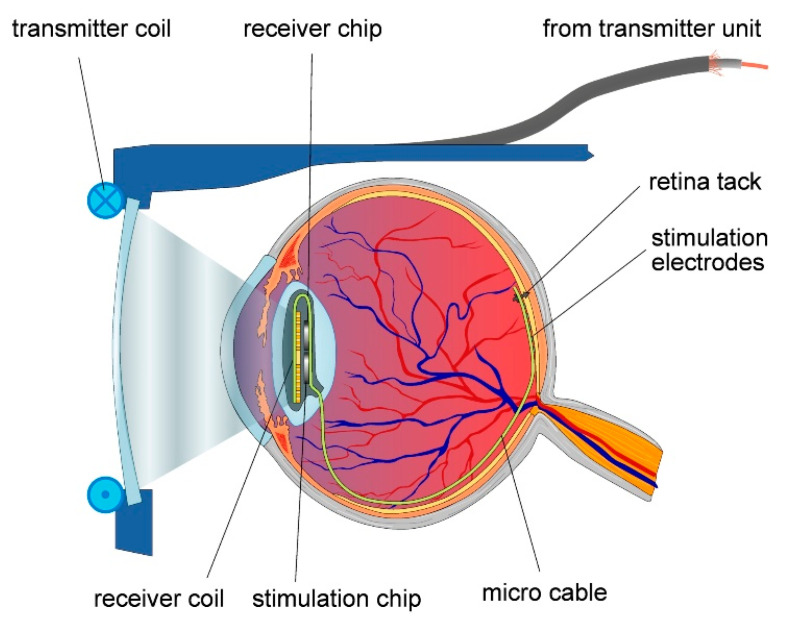
Retinal implant [75]. Reproduced with the kind permission of Prof. Wilfried Mokwa, RWTH Aachen.

More details of the device around and in the eye are given in Figure 16. The basic idea is similar to that of the cochlear implant. In this case an external camera sends a signal to the implant, which stimulates the nerves at the back of the eye. Just like with the cochlear implant, there have been ideas to connect directly into the optical nerve, or use probes directly into the brain.

As with the cochlear implant there is still a limitation in the number of electrodes. The company IRIS^®^ Bionic Vision System has 150 electrodes. Although this does not give a high-quality image, it does allow a blind person sufficient image quality to function and navigate around objects. This work has shown the feasibility of the approach, and work is continuing to improve the image quality [76,77], including increasing the area of the electrode array and increasing the density of electrodes [78,79]. Another approach is to implant arrays of the probe directly into the optical cortex at the back of the brain [80]. This could, potentially, give a good quality image. However, issues with this approach remain in terms of the long-term stability and the risk of probes breaking.

### 6.4. Glucose Measurement

**Figure 17:**
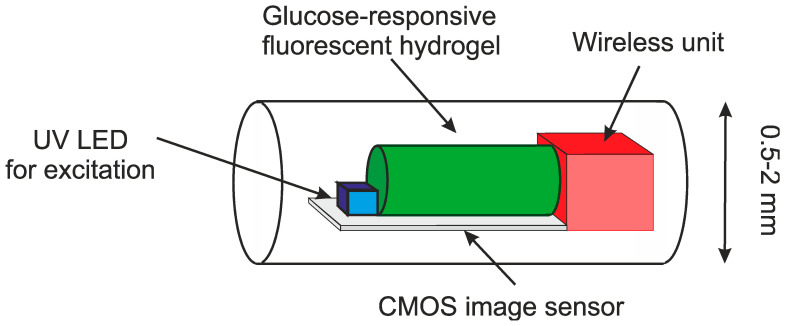
Bio-inspired implantable glucose sensor using a fluorescent hydrogel [81].

As mentioned above, the traditional method for measuring glucose is a blood sample. A chemical analysis can then be performed to establish the glucose levels. Advances in microsystems have led to the use of smaller samples. More recently, skin electrodes have been developed. These work on measuring just under the epidermis with microneedles of electrodes on the skin. The next step is total implantation and wireless communication. One such example is given in Figure 17 [81]. The device is implanted just under the skin and the resulting fluorescence can then be detected in the unit and transmitted to the external device. The external device could be a mobile phone, or a dedicated unit. If a device is controlled by an external unit through magnetic coupling, an internal battery is not required. However, the long-term safety and stability of the device remains important. Some research suggests including an implanted pump with an insulin source. To have a practical implanted pump we need to have the device implanted for a long period. This presents important issues for biocompatibility and safety.

**Figure 18:**
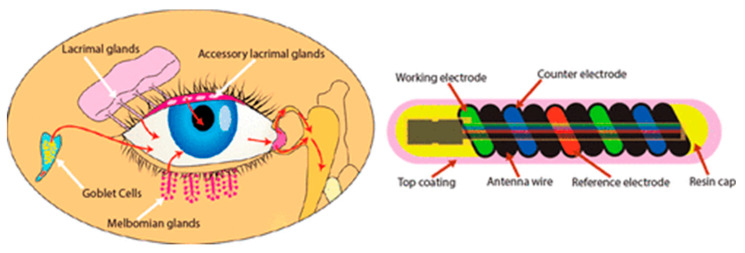
Glucose sensor for the eye [86]. Reproduced with the kind permission of Noviosense (NL).

Glucose sensors have also been made for implantation in the eye and bladder [82,83,84,85,86,87]. This is a good measure of glucose in the body, but there is a delay in the change compared to blood. An example of a glucose sensor for the eye is given in Figure 18 [86]. This approach is less invasive than the implanted approach. The hollow core allows the device to be flexible and fit into the environment. This part contains the communication. Inside the coil is a glucose sensitive hydrogel.

The same issue of delay applies for devices in the bladder. Both of these approaches are excellent ways to measure glucose, although with the slight delay compared to blood measurements. In this case the device should be fixed to the wall of the bladder and it is important that the device does not result in any bladder infections.

### 6.5. Brain Implants

**Figure 19:**
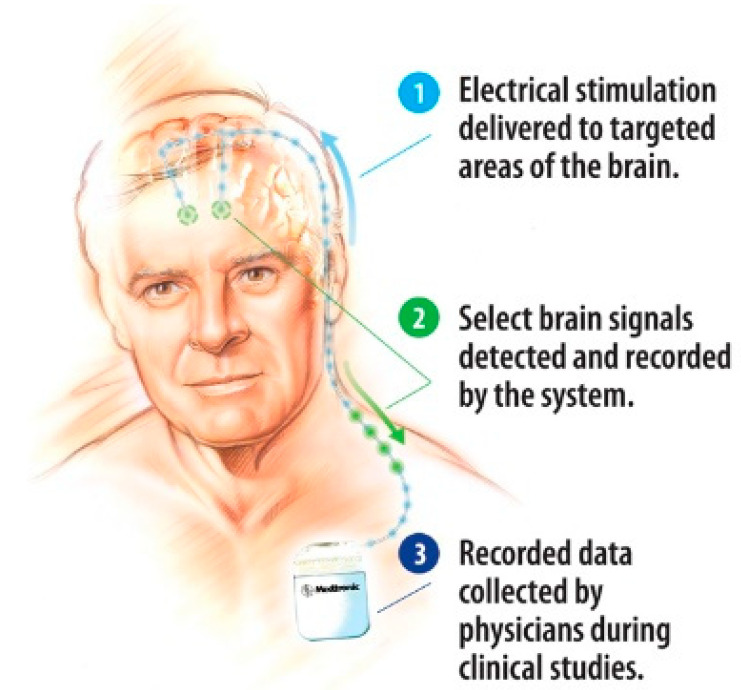
General view of a deep brain implant system [88].

Deep brain implants use, in general, a similar technique to the pacemaker, with a unit implanted in the chest and with cables leading to the probe, which in turn brings the signal to the point of interest in the brain. These electrodes can disrupt, or cancel unwanted signals from the brain. They can be used to block epilepsy, the shake due to Parkinson’s disease and many other conditions. Such a system, developed by Medtronic, is shown in Figure 19. It is also important that the brain accepts the signal and thus the format of the signal and varying the signal can be very important.

Electrodes have also been used to measure and stimulate in the outer layers of the brain. Present research is moving towards a single unit attached to the brain, removing the need for a unit in the chest. With Parkinson’s there is also a lower level of dopamine. The levels of dopamine can be measured with electrodes in the brain [89]. There have also been probes developed to stimulate the visual cortex to restore sight to the blind [90]. In this case, rather than sending the signal from a camera to the retina, the signal is fed to the visual cortex via a probe array.

Further advances in electronics and miniaturization are leading to the idea of bidirectional interfaces with the brain, positioned on the surface of the brain. This requires the measurement, signal processing and stimulation to be performed in compact devices and active electrodes [91].

## 7. Micropumps

**Figure 20:**
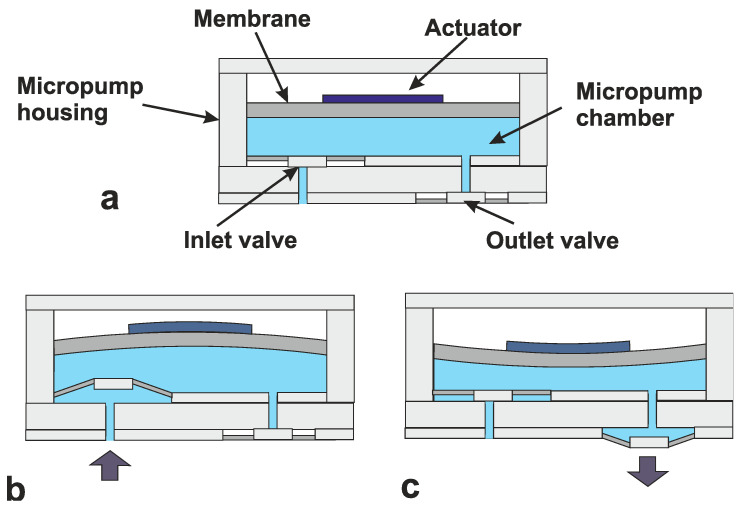
Three basic cycles in micropumps (**a**) initial starting point, (**b**) increased chamber volume and (**c**) decreased chamber volume [96].

The idea of micropumps date back to the 1970s [92], but real interest came with the first micromachined micropump from Jan Smits and Harald van Lintel [93]. The ability to accurately pump extremely small volumes saw applications grow in the biochemical field. This meant that considerably smaller samples could be used, reducing costs and increasing analysis speed. Pumps can be mechanical and non-mechanically driven [94,95]. Most mechanical pumps use a membrane to increase and decrease the pressure in a chamber. The membrane can be activated using a number of actuators including piezoelectric, thermal magnetic and electrostatic. The created pressure differences cause the pumping mechanism. This sequence is shown in Figure 20 [96]. In this figure, Figure 20a shows the initial starting point. The actuator is activated increasing the volume of the chamber (Figure 20b) thus sucking fluid in through the (passive) inlet valve. By releasing (or reversing) the actuator, the volume is decreased, which increases the pressure and opens the outlet valve (Figure 20c). This basic cycle works with all the actuator techniques. In some cases, a double chamber is used. This can help reduce back-flow, but the principle is the same.

With chronic complaints it can be greatly beneficial to implant a pump with a capsule of medicine. This will enable regular small doses and, in some cases, a more concentrated dose directly to where it is needed [97,98,99,100].

Combining these pumps with glucose sensors could lead to a fully implanted artificial pancreas [101]. As with all implants there remain issues of safety and long-term stability. For micropumps we also need to consider the safety of the storage of liquid to be pumped.

## 8. Power and Communication

**Figure 21:**
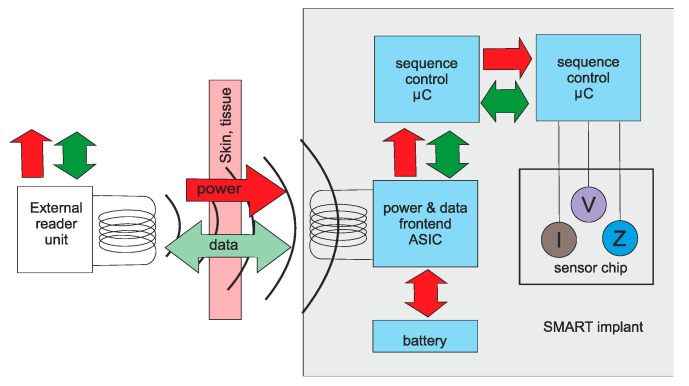
Magnetic coupling for medical implants [102].

With surface devices, power and communication are generally not an issue, since there is direct access to the device. Additionally, for catheter devices the power and communication can be sent down the catheter itself. Once a device is completely implanted into the body, power and communication become important considerations. Pacemakers generally use batteries encased in the main unit. Battery technology allows these to operate for many years. Other options include magnetic coupling [102], as shown in Figure 21.

**Figure 22:**
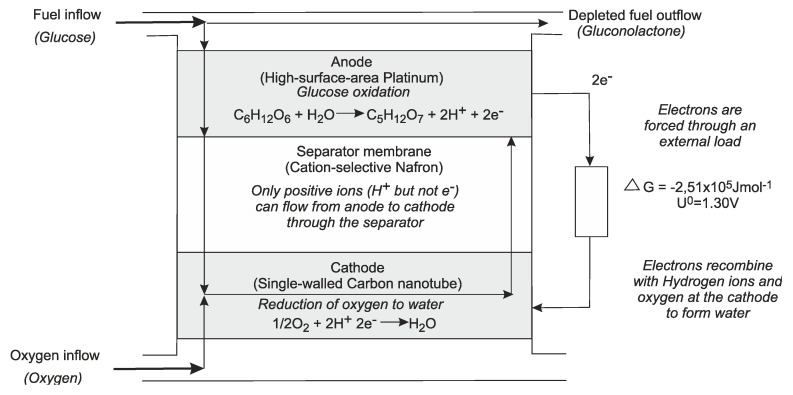
Fuel cell using glucose [107].

Energy scavenging can also be used. Piezoelectric devices work on the dynamic force to generate a voltage, other options are flow or temperature differences [103,104,105,106,107]. Energy can also be generated from the glucose in the body, in the form of a fuel cell [106,107]. An example of this is shown in Figure 22.

Communication is generally achieved using a wireless unit. These usually only require a range of less than 1 m since the external unit is worn by the user, or next to the patient in a hospital. There are restrictions on the wavelengths used due to regulations and also the body’s absorption. A typical range is 401–406 MHz [108]. Furthermore, due to the limitations on power, the system should be optimized to transmit only essential information.

## 9. Discussion and Conclusions

Microsystems have played a major role in medical development. In biochemical analysis they have allowed miniaturization leading to faster results with a less biological sample. In the field of in-vivo devices, miniaturization has led to many new applications. The development of flexible devices means they can be molded to the shape of the skin, or implanted into/onto organs. The catheter has been used for centuries, but in the 20th century, cardiac catheterization was born. This led to a minimally invasive technique allowing measurement and treatment to be performed with minimal damage to healthy tissue. Keyhole surgery using miniaturized instruments has also led to less damage to healthy tissue and faster recovery times. Fully implanted systems have allowed monitoring, and in some case treatment, for a short or long period to be performed. Long term implants can be used for monitoring and also for restoring senses. These include cochlear implants for the deaf and retinal implants for the blind and pacemakers. For completely implanted systems it is important to consider the powering of the system. There are many options for this including batteries, magnetic coupling and energy scavenging. For all devices in contact with living tissue, it is essential that the biocompatibility and safety are shown before the device can be used in medical practice.
